# An Evaluation of Epidemiological and Reporting Characteristics of Complementary and Alternative Medicine (CAM) Systematic Reviews (SRs)

**DOI:** 10.1371/journal.pone.0053536

**Published:** 2013-01-14

**Authors:** Lucy Turner, James Galipeau, Chantelle Garritty, Eric Manheimer, L. Susan Wieland, Fatemeh Yazdi, David Moher

**Affiliations:** 1 Ottawa Hospital Research Institute, Clinical Epidemiology Program, Ottawa, Ontario, Canada; 2 Center for Integrative Medicine, University of Maryland School of Medicine, Baltimore, Maryland, United States of America; 3 Department of Epidemiology and Community Medicine, University of Ottawa, Ottawa, Ontario, Canada; Queen Elizabeth Hospital, Hong Kong

## Abstract

**Background:**

Systematic reviews (SRs) are abundant. The optimal reporting of SRs is critical to enable clinicians to use their findings to make informed treatment decisions. Complementary and alternative medicine (CAM) therapies are widely used therefore it is critical that conduct and reporting of systematic research in this field be of high quality. Here, methodological and reporting characteristics of a sample of CAM-related SRs and a sample of control SRs are evaluated and compared.

**Methods:**

MEDLINE® was searched to identify non-Cochrane SRs indexed from January 2010 to May 2011. Control SRs were retrieved and a search filter was used to identify CAM SRs. Citations were screened and publications that met a pre-specified definition of a SR were included. Pre-designed, standardized data extraction forms were developed to capture reporting and methodological characteristics of the included reviews. Where appropriate, samples were compared descriptively.

**Results:**

A total of 349 SRs were identified, of which 174 were CAM-related SRs and 175 were conventional SRs. We compared 131 CAM-related non-Cochrane SRs to the 175 conventional non-Cochrane reviews. Fifty-seven percent (75/131) of CAM SRs specified a primary outcome compared to 21% (37/175) of conventional sample reviews. Reporting of publication bias occurred in less than 5% (6/131) of the CAM sample versus 46% (80/175) of the conventional sample of SRs. Source of funding was frequently and consistently under-reported. Less than 5% (11/306) of all SRs reported public availability of a review protocol.

**Conclusion:**

The two samples of reviews exhibited different strengths and weaknesses. In some cases there were consistencies across items which indicate the need for continued improvements in reporting for all SR reports. We advise authors to utilise the PRISMA Statement or other SR guidance when reporting SRs.

## Introduction

Systematic reviews (SRs) are a prominent and established component of evidence-based health care. On average, 11 new reviews are published daily [1]. As with all research, the value of a SR depends on how it was conducted and reported. The reporting quality of SRs varies [2], limiting readers’ ability to assess the strengths and weaknesses of reviews [3]. Poorly conducted and/or reported SRs may limit their usefulness for practice guideline developers and other stakeholders, such as policy makers.

In 2007, Moher et al. examined the epidemiological and reporting characteristics of a cross-section of 300 SRs indexed in MEDLINE® in November of 2004 [4]. The authors noted: 40.7% of reviews did not report a source of funding; only 66.8% reported conducting some form of risk of bias assessment; and only 23.1% reported assessing publication bias. Just over half (53.7%) of evaluated reviews reported combining their results statistically, of which 91.3% assessed consistency across pooled studies. Only 17.7% were reported to be updates of SRs. No reviews reported a protocol registration number.

The prevalence of Complementary and Alternative Medicine (CAM) use in the general population is considerable [5]. There are differences across surveys reporting global prevalence estimates of overall use of CAM which can be largely explained by different definitions of CAM within the various surveys. In 2000, a SR of surveys conducted to examine the prevalence of CAM use among general populations in countries worldwide found that a substantial proportion of the surveyed populations used CAM. However comparisons, both across countries and within countries, was difficult because of differences in definitions of CAM, differences in the reference time period for the use of these therapies, differences in study designs, and other methodological differences between surveys [6], [7]. One recent estimate from a 2007 NIH survey suggests that 38.3% of American adults use some form of CAM [5]. Regardless of the exact figure, the use of CAM treatments is prevalent in the general population. Therefore, it is critical that CAM research in this area, like all health research, adhere to high conduct and reporting standards in order to enable knowledge users to interpret report findings with confidence.

Information regarding deficiencies in the quality or reporting of specific aspects of SRs enables researchers to target methodological aspects of review conduct and reporting that can be improved with the aim of producing higher quality research. This report therefore evaluates and compares the methodological and reporting characteristics of two cross sectional MEDLINE® samples of SRs; CAM specific SRs and a sample of SRs across a variety of clinical topics. We also draw comparisons with the Moher 2007 paper [4] from which this evaluation was methodologically derived.

## Methods

### Sample Criteria

Two cross sectional samples of SRs published outside of the Cochrane Library have been evaluated. The first was comprised of SRs indexed from January 2010 to May 2011 pertaining to Complementary and Alternative Medicine, henceforth referred to as “CAM”, as defined, categorized and operationalized by the Cochrane CAM Field [8]. The second sample, over the same time period, consisted of a cross sectional sample of SRs published in core clinical journals [9], henceforth referred to as “control”.

### Eligibility Criteria

**Figure 1:**
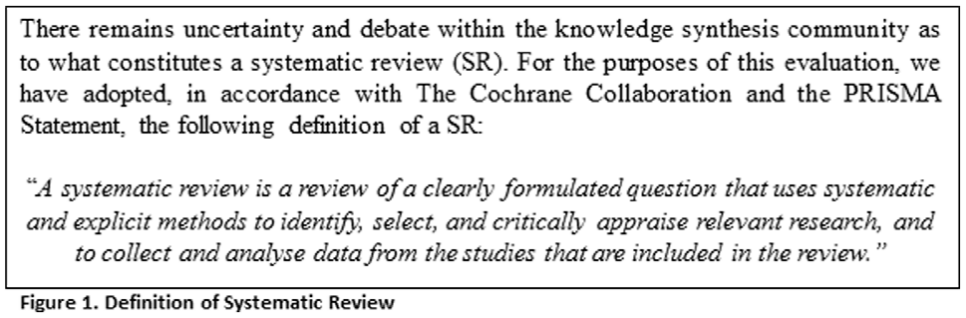
Defining a ‘systematic review’.

To be eligible for inclusion, articles for both samples had to meet the following definition of a SR [10], [11]: search at least one database; provide a description of at least one eligibility criterion; and report the critical appraisal of included studies (Figure 1). Any type of SR was eligible (e.g., comparative effectiveness, prognostic, diagnostic), overviews of SRs were not eligible for inclusion. Unpublished SRs, including grey literature, were not included and SRs were not restricted by language of publication.

### Electronic Search Strategy

We conducted two independent electronic searches to identify both samples. For the CAM sample, we searched MEDLINE® via Ovid using an unpublished filter iteratively developed by a group of information specialists on behalf of the Canadian Agency for Drug and Technologies in Health [12]. We then conducted the same search of MEDLINE® via Ovid, without filtering for CAM SRs, to identify control SRs [Appendix S1]. Due to the volume of literature, the search was limited to identify reports of SRs indexed between January 2010 and May 2011, inclusive. The search for the control sample was limited to core clinical journals [9].

### Study Selection

Retrieved citations were screened based on inclusion criteria for a SR using online review software, DistillerSR® [13]. Title and abstract screening was conducted by liberal acceleration (i.e., two reviewers needed to independently exclude a record; only one reviewer needed to include a record) and subsequent full text articles were retrieved and screened independently by two of four reviewers. Any disagreements were discussed and remaining conflicts were resolved by third party consensus. When full text screening of both samples was complete, a random sample of control SRs was generated by SAS, Version 9.1 [14], matching the total number of eligible CAM SRs. Translators, trained in epidemiology or biostatistics, assessed the eligibility of the non-English language studies identified.

### Data Collection and Analysis

Data were collected for both samples using a standardized form of 49 questions (available upon request). Items for data collection were determined a priori based on the Moher 2007 items [4]. Pilot testing of the data extraction form was conducted to ensure consistency. Data extraction was completed and a 10% random sample of SRs was extracted independently in duplicate to assess accuracy within both samples. Extractors discussed and resolved all disagreements in order to achieve consensus.

Data were collected regarding three review components: epidemiological, descriptive and reporting characteristics of SRs. Epidemiological characteristics included, for example, the number of authors per review, country of corresponding author, and review ICD-10 categories. Descriptive characteristics of the assessed SRs included, for example, the use of data management software, the number of included studies, and the use of reporting guidelines. Using the Preferred Reporting Items for Systematic Reviews and Meta-Analyses (PRISMA) checklist as a template [3], reporting characteristics were assessed for inclusion of items such as eligibility criterion, description of search strategy, data extraction, results, analysis, and source of funding.

All analyses are descriptive, with data summarized using frequency and percentage, or median and inter-quartile range (IQR) of SRs for both samples.

## Results

### Search Results and Included Trials

#### CAM SRs

**Figure 2:**
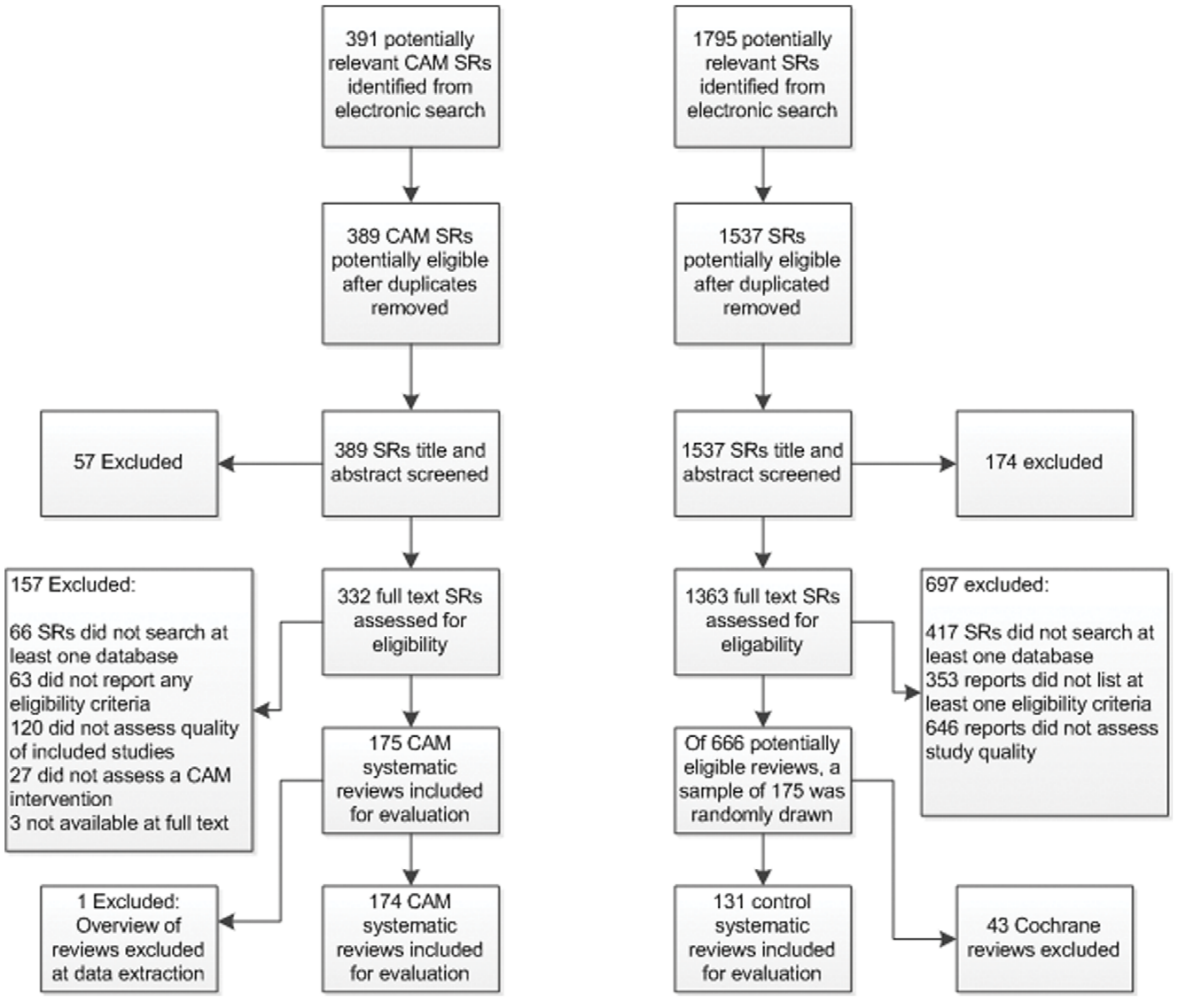
Flow diagram of included systematic reviews.

Electronic searching yielded a total of 389 unique records of which 57 were excluded at title and abstract screening. A total of 174 SRs were included in the CAM sample. Of the 174 CAM sample SRs evaluated, 43 were identified as reviews published in the Cochrane Library. As Cochrane reviews follow specialized, detailed and consistent methodology and reporting guidelines and as a result may differ in quality [15], [16]. The CAM SRs sample was adjusted to exclude Cochrane Reviews in order to ensure comparability with the control sample. Results of a total of 131 CAM SRs are reported of which 6 were non-English language (Figure 2).

#### Control SRs

Electronic searching yielded a total of 1,537 possibly relevant citations for the control sample of which 174 reports were excluded during title and abstract screening. Of 1,363 SR reports reviewed at full text, 697 were excluded and the remaining 666 reports were eligible for inclusion. Of those eligible a random sample of 175 SR reports was included (Figure 2).

### Epidemiology of Systematic Reviews

The median journal impact factor (2010) was lower for the CAM sample [median (IQR) 2.19 (1.50, 3.40)] across 93 journals compared to the control sample [median (IQR) 5.39 (3.40, 10.78)] across 61 journals. Overall, CAM SRs had fewer authors cited than SRs in the control sample, with 36% (47/131) of CAM reviews and 23% (41/175) of the control sample authored by 2–3 persons, compared to 42% (55/131) of CAM SRs and 54% (94/175) of control SRs with 4–6 authors. CAM reviews were more evenly distributed over corresponding authors’ country, with 29% (38/131) of authors from one of 17 other countries in contrast to, 29% (52/175) of control SRs whose corresponding authors were based in the United States. Corresponding authors with South Korean and Chinese affiliations differed between the CAM and control samples, with 10% (13/131) of reviews in the CAM sample with South Korean authors versus none of the control SRs, and 13% (17/131) Chinese corresponding authors in the CAM sample compared to 6% (11/175) in the control group.

The six most common ICD-10 categories for SRs were similar across both samples, however, there were notably fewer (0.76%, 1/131) CAM reviews focusing on pregnancy, child birth and purperium compared to the control sample (13.71%, 24/175). Almost all CAM SRs focused primarily on treatment (93.13%, 122/131), considerably more than the control sample (54.29%, 95/175). None of the CAM SRs focused on prevention, diagnosis or prognosis, while 7% (9/131) of reviews assessed either prevalence of use, education, or overall health, which we categorized as ‘other’ in our data extraction (Table 1).

**Table 1:** Epidemiology of Systematic reviews.

		CAM Group	Control Group	Moher 2007 [4]
Category	Characteristics	n (%), N = 131	n (%), N_1_ = 175	n (%), N = 300
**Total Number of Journals**		93	61	132
**2010 Journal Impact Factor, Median (IQR)**		2.19 (1.50, 3.40)	5.39 (3.40, 10.78)	
**Number of authors**	1	6 (4.58)	1 (0.57)	24 (8.0)
	2–3	47 (35.88)	41 (23.43)	125 (41.7)
	4–6	55 (41.99)	94 (53.71)	128 (42.7)
	≥7	23 (17.58)	39 (22.29)	23 (7.7)
**Country of Corresponding Author**	Australia	9 (6.87)	6 (3.43)	31 (10.30)
	Austria	1 (0.76)	3 (1.71)	
	Canada	11 (8.40)	30 (17.14)	28 (9.3)
	China	17 (12.80)	10 (5.71)	
	France	–	4 (2.29)	
	Germany	5 (3.82)	5 (2.86)	10 (3.3)
	South Korea	13 (9.92)	0 (0.00)	
	The Netherlands	6 (4.58)	11 (6.29)	17 (5.7)
	UK	19 (14.50)	27 (15.43)	76 (25.3)
	US	21 (16.03)	52 (29.14)	68 (22.7)
	Other^a^	38 (29.01)	27 (15.43)	60 (20.0)
**Common ICD-10 Categories**	Diseases of the circulatory system	15 (11.45)	30 (17.14)	33 (11.0)
	Diseases of the genitourinary system	8 (6.11)	8 (4.57)	
	Diseases of the musculoskeletal system and connective tissue	31 (23.66)	18 (10.29)	
	Malignant neoplasms	8 (6.11)	14 (8.00)	22 (7.3)
	Mental and behavioural disorders	16 (12.21)	19 (10.86)	40 (13.3)
	Pregnancy, childbirth and the puerperium	1 (0.76)	24 (13.71)	21 (7.0)
**Primary Focus**	Treatment	122 (93.13)	95 (54.29)	21.3 (71.0)
	Prevention	0 (0.0)	27 (15.43)	
	Prognosis	0 (0.0)	24 (13.71)	23 (7.7)
	Diagnosis	0 (0.0)	13 (7.43)	
	Other^b^	9 (6.87)	16 (9.14)	46 (15.3)

a **Moher 2007** [**4**] **:** 30 countries <10 reviews/country. **Control Group:** India, Norway, Sweden, Belgium, Brazil, Denmark, Greece, Israel, Italy, Poland, Singapore, Spain, Switzerland. **CAM Group:** Japan, New Zealand, Thailand, Belgium, Chile, Denmark, Hong Kong, Ireland, Italy, Malaysia, Nigeria, Oman, Peru, Saudi Arabia, South Africa, Spain, Taiwan.

b Methodological, educational, prevalence of use, overall health effects and mindfulness.

### Descriptive Characteristics of Systematic Reviews

Fewer CAM SRs were updates of original reviews (5.34%, 7/131) compared to control SRs (10.29%, 18/175). The median number of included studies was similar across both samples, as were the number of included participants. Likewise, the number of SRs considering cost-effectiveness analysis was comparable in the CAM and control samples [3% (4/131) and 2% (3/175), respectively].

The reported use of free and commercially available SR software in both samples was low, less than 3% (4/131) for CAM reviews and less than 5% (8/175) of control reviews. Fewer CAM SRs reported using any reporting guidelines (23.67%, 31/131) compared to the control sample SRs (50.86%). However, substitute use of reporting guidance, such as using a reporting guideline for RCTs (i.e. CONSORT [17]) instead of one for SRs (i.e. PRISMA [3], MOOSE [18]), was higher in the control sample of SRs (18.86%, 33/175) versus the CAM SRs, where no misuse or substitution was identified. Meta-analysis was less frequently performed in CAM reviews, with pooled effects reported in less than 50% (65/131) of CAM SRs versus 75% (132/175) of control SRs. The median number of meta-analyses per review were similar for the CAM sample [median (IQR), 4 (3, 9)] and the control sample [median (IQR), 7 (4, 14)]. However, there were far more CAM reviews compared to control reviews reporting only 2 studies in their largest meta-analysis ([11% (7/131)] versus) <1% (1/175), respectively]). Random effects models were more frequently used across all reviews for meta-analyses, while 19% (12/131) of CAM SRs and 17% (22/175) of control SRs reported using both random and fixed effects models. Almost 10% (6/131) of CAM reviews and 5% (7/175) of control reviews did not report which model(s) used when running meta-analyses (Table 2).

**Table 2:** Descriptive Characteristics of systematic reviews.

		CAM Group	Control Group	Moher 2007 [4]
Category	Characteristics	n (%), N = 131	n (%), N_1_ = 175	n (%), N = 300
**Type of Interventions** c	Pharmacological	34 (29.60)	57 (32.57)	142 (47.3)
	Non-Pharmacological	94 (71.75)	61 (34.95)	113 (37.7)
	No Intervention	3 (2.29)	57 (32.57)	42 (14.0)
**Update of a previous review**	Updated	7 (5.34)	18 (10.29)	53 (17.7)
**Number of included studies, Median (IQR)**		14 (8, 28)	18 (10.25, 33.75)	16 (7, 30)
**Number of participants, Median (IQR)**		1013 (475.25, 2033.75)	2815 (1111, 8460)^a^	1,112 (322–3,750)
**Economics considered**	Cost-effectiveness analysis conducted	4 (3.05)	3 (1.71)	61 (24.0)
	No	124 (94.65)	172 (98.29)	193 (76.0)
**Cochrane Review**	Yes (includes co-publications)	3 (2.29)	2 (1.16)	–
	No	128(97.70)	173 (98.86)	–
**Data Management Software**	Used^d^	4 (3.05)	8 (4.57)	–
	Not used/Not reported	127 (96.94)	167 (95.43)	–
**Reported Use of Reporting Guidelines**	None	100 (76.33)	86 (49.14)	–
	PRISMA [3]	3 (2.29)	21 (12.00)	–
	QUOROM [29]	7 (5.34)	13 (7.43)	–
	MOOSE [18]	0 (0.0)	16 (9.14)	–
	Substitute use^e^	0 (0.0)	33 (18.86)	–
	Other^f^	21 (16.03)	6 (3.43)	–
**Quantitative Analysis**		**65 (49.61)**	**132 (75.43)**	**161 (53.7)**
**Number of pooled effects** g **, Median (IQR)**		4 (3, 9)	7 (4, 14)	–
**Number of studies in largest meta–analysis**	2	7 (10.93)	1 (0.76)	**–**
	3–5	20 (31.25)	27 (20.45)	–
	6–8	14 (21.87)	22 (16.67)	–
	9–11	8 (12.50)	20 (15.15)	–
	12–25	9 (14.06)	41 (31.06)	–
	26–50	4 (3.05)	14 (10.61)	–
	50–100	1 (0.76)	3 (2.27)	–
	100+	0 (0.0)	1 (0.76)	–
**Unit of measure for the primary outcome(s)**	Difference in Means	15 (23.43)	30 (22.73)	–
	Standardised Mean Difference	17 (26.56)	14 (10.61)	–
	Risk Ratio	18 (28.12)	29 (21.97)	–
	Odds Ratio	7 (10.93)	39 (29.55)	–
	Risk Difference	2 (3.15)	2 (1.52)	–
	Hazard Ratio	0 (0.0)	12 (9.10)	–
	Likelihood Ratios	0 (0.0)	10 (7.58)	–
	PPV, NPV	0 (0.0)	4 (3.03)	–
	Unclear	2 (3.15)	0 (0.00)	–
**Model applied**	Random Effects	37 (57.81)	67 (50.76)	–
	Fixed Effects	**9 (14.06)**	17 (12.88)	–
	Both	12 (18.75)	22 (16.67)	–
	Not Reported	6 (9.37)	7 (5.30)	–

c It should be noted that where CAM reviews are pharmacological pertains to reviews which include a CAM and conventional intervention.

d SRS [13], RevMan [32], Endnote [33], GRADEpro [34], Refworks [35].

e Substitute use defined as using CONSORT [17], STROBE [36], STRICTA [30] and GRADE [37] for reporting SRs.

f Specifically referred to as reporting guidance, Cochrane Handbook or named Cochrane review group [10], STRICTA [30], GRADE [37], Centre for evidence-based medicine guidelines at the University of Oxford [38], NICE Guidance [39], Cooper’s 5-stage model, Guidelines from the Philadelphia panel classification system [40], AHRQ guidance [41].

g Synthesis had to include more than one study and estimates reported both in the text and as a figure were only included in the count once.

### Reporting Characteristics of Systematic Reviews

Over 20% (28/131) of CAM SRs and 16% (28/175) of control SRs did not use the terms “systematic review” or “meta-analysis” the title of the review report (Table 3). Fewer CAM reviews were described as a “meta-analysis” in the title and abstract [44% (57/131) versus 60% (105/175) of control SRs]. Of those described as a meta-analysis in the title, 22.81% (30/131) of CAM SRs versus 1% (2/175) of the control SRs did not report pooled estimates of effect.

**Table 3:** Reporting Characteristics of systematic reviews.

Category	Subcategory	Group	CAM Group n (%), N = 131	Control Group n (%), N = 175	Moher 2007 [4] N (%), N = 300
	Use of term “systematic review” in title		97 (74.04)	116 (66.29)	150 (50.0)^h^
	Use of term “meta-analysis in title”		57 (43.51)	105 (60.00)	–
	Neither term reported		28 (21.34)	28 (16.00)	150 (50.0)
	Protocol Mentioned	Total Reported	7 (5.34)	25 (14.29)	139 (46.3)
		Publically Available	3 (2.29)	8 (4.7)	–
**Eligibility Criteria**	Subject to study design	No restrictions	11 (0.76)	50 (28.57)	–
		RCT	84 (64.12)	58 (33.14)	176 (60.1)
		RCT and Others	20(15.26)	39 (22.29)	–
		Observational Case Controlled	0 (0.00)	10 (5.71)	21 (7.2)
		Observational	0 (0.00)	12 (6.86)	14 (4.8)
		Prospective studies	5 (3.81)	5 (2.86)	–
		Other/Unclear	4 (3.05)	1 (0.57)	89 (29.67)
	Subject to publication status	Yes, published and unpublished	20 (15.26)	33 (18.86)	123 (41.0)
		Yes, unpublished only	0 (0.00)	0 (0.00)	0 (0.00)
		Yes, published only	52 (39.69)	69 (39.43)	68 (22.7)
		Not reported/Unclear	55 (41.98)	73 (41.71)	109 (36.3)
	Subject to language of publication	English Only	28 (21.37)	79 (45.14)	49 (16.3)
		Specified mix	20 (15.26)	24 (13.71)	6 (2.0)
		No restrictions	64 (48.85)	63 (36.00)	110 (36.7)
		Not reported	15 (11.45)	9 (5.14)	134 (44.7)
**Search**	Number of databases searched, Median (IQR)		6 (4, 7)	3 (2, 5)	3 (2, 5)
	Medline or EMBASE searched		89 (67.93)	172 (98.29)	–
	Non-electronic methods of searching reported		87 (66.41)	154 (88.00)	–
	reporting of search years of coverage	Yes	113 (86.25)	61 (34.86)	208 (69.3)
		Partially reported	13 (9.92)	110 (62.86)	49 (49 (16.3)
		No	5 (3.81)	4 (2.29)	43 (14.33)
	Search terms reported	No search terms reported	12 (9.16)	13 (7.42)	37 (12.3)
		Full search strategy as appendix or link	37 (28.24)	23 (13.14)	132 (44.00)
		Keywords, MeSH index terms and/or free text reported and/or topics	82 (62.59)	119 (68.00)	128 (42.67)
**Data Extraction**	Specified one or more primary outcome		75 (57.25)	37 (21.14)	143 (51.1)
	Methods of screening	Two reviewers in duplicate	55 (41.98)	92 (52.57)	–
		By two of many	6 (4.58)	8 (4.57)	–
		One reviewer only	4 (3.05)	8 (4.57)	–
		Not reported	63 (48.09)	67 (38.29)	–
	Methods of data extraction	Two reviewers in duplicate^i^	79 (60.30)	100 (57.14)	–
		By two of many	5 (3.81)	14 (8.00)	–
		Sample verification	2 (1.52)	6 (3.43)	–
		One reviewer only	4 (3.05)	5 (2.86)	–
		Not reported	41 (31.29)	50 (28.57)	–
	Means of quality assessment	Risk of Bias Tool [19] or modification of	36 (27.48)	30 (17.14)	–
		Jadad [42] or modification	41 (31.30)	24 (13.71)	–
		Newcastle-Ottawa Scale [43]	1 (0.76)	10 (5.71)	–
		Reporting Guideline	5 (3.81)	13 (7.43)	–
		Did not report tool	5 (3.81)	9 (5.14)	–
		Self-developed	5 (3.81)	19 (10.86)	–
		Other^j^	66 (50.38)	70 (40.00)	–
**Results**	A description of review flow	None	9 (6.87)	14 (8.00)	92 (30.7)
		Partial, text and/or table	10 (7.63)	11 (6.29)	99 (33.0)
		Complete, text and/or table	32 (24.43)	21 (12.00)	106 (35.3)
		Complete, PRISMA flow like diagram	14 (10.69)	96 (54.86)	20 (6.7)
		Complete, PRISMA flow like diagram and in text and/or table	66 (50.38)	35 (20.00)	–
	Reasons for exclusion	Fully reported	28 (21.37)	139 (79.43)	144 (48.0)
		Partially reported	5 (3.81)	16 (9.14)	119 (39.7)
		None	8 (6.10)	20 (11.42)	50 (16.7)
	Grey literature^k^ included		29 (22.13)	37 (21.14)	132 (44.0)
	Consistency	Formally assessed	59 (90.76)	117 (66.86)	147 (49.0)
		Qualitatively assessed	1 (0.76)	7 (4.00)	51 (17.0)
		Not assessed/Not reported	5 (7.69)	51 (29.14)	102 (34.0)
	Common methods for assessing consistency	I^2^	50 (38.16)	95 (71.97)	–
		Cochrane Q/Chi^2l^	32 (24.42)	32 (24.24)	–
		Visual Inspection	1 (0.78)	2 (1.51)	–
		Tau^2^	13 (9.92)	1 (0.76)	–
		L’Abbe Plot	0 (0.00)	2 (1.51)	–
	Selective reporting was explicitly assessed		21 (16.03)	43 (24.57)	–
	Assessment of publication bias reported		6 (4.58)	80 (45.71)	92 (31.3)
	Common methods for assessing publication bias	Funnel Plot	2 (1.52)	79 (45.14)	–
		Egger’s Test	1 (0.76)	7 (4.00)	–
		Regression	1 (0.76)	35 (20.00)	–
		Begg Test	1 (0.76)	3 (1.71)	–
		Trim and Fill	0 (0.0)	11 (6.29)	–
	Impact of assessment discussed in results		28 (23.14)	81 (46.29)	–
**Discussion**	Limitations discussed		107 (81.67)	157 (89.71)	–
**Other**	Source of Funding	Non-Profit	27 (20.61)	63 (36.00)	144 (48.0)
		For profit	0 (0.0)	6 (3.43)	7 (2.3)
		Author specified no funding	46 (35.11)	28 (16.00)	3 (1.0)
		Mixed	1 (0.76)	0 (0.00)	19 (6.3)
		Not Reported	36 (27.48)	78 (44.57)	127 (42.4)

h Reported as either “systematic review” or “meta-analysis”.

i Includes 100% verification.

j **Control Group:** GRADE [37]; AHRQ Guidance [41]; Egger’s tool [44]; Downs and Black [22]; Zaza et al. [23]; publication bias only assessed; International Society of Pharmacoeconomics and Outcomes Research [45]; The Delphi list [46]; US Preventative Services Task Force criteria [47]; Cho and Bero [48]; Sauerland [49]; America academy of neurology [50]; PEDro [51]; COREQ [52]; West [53]; Schulz’s Allocation concealment [54]; outcome reporting bias only; Centre of evidence-based medicine at the University of Oxford [55]; MINORS [24]; DTA assessment; “assessed based on study design”; labelled sensitivity analysis as quality assessment; adjusted analysis by characteristics calling it quality assessment; **CAM Group**: GRADE [37]; NICE [40]; EPC based [41]; Downs and Black [22]; Delphi list [46]; PEDro [51]; Allocation concealment [54]; Centre of Evidence-Based Medicine at the University of Oxford [55]; McMaster Quality Assessment Scale of Harms (McHarm) [56]; Oxman and Guyatt [57]; Centre for reviews and dissemination [58]; MINORS [24]; CASP [59]; Scottish Intercollegiate Guidelines Network [60]; Stetler’s Evidence Ranking system [61]; Tulder Score [62]; MINORS [24]; Wilson and Lawrence Scores; RAC; Ostello.

k Gray Literature searching refers to systematic review search methods to identify primary studies which are not identified via standard searching methods [63].

l Independent of I^2^.

#### Eligibility criteria and search

Less than 5% of all SRs reported public availability of a review protocol [2.29% (3/131) of CAM SRs versus 4.70% (8/175) of control SRs]. CAM SRs were more likely than control SRs to restrict eligibility of primary studies to RCTs [64% (84/131) versus 33% (58/175), respectively], when adjusting for primary review focus, treatment-only control 44.21% (42/95) of SRs were restricted to include RCTs only. Less than 20% of reviews in both samples (15% of CAM SRs and 19% of control SRs) considered both published and unpublished literature for inclusion. CAM reviews were less likely to restrict eligibility by language of publication, with 22% (28/131) restricted to English versus 45% (79/175) of the control sample reviews. The median number of electronic databases searched for CAM reviews was higher than that for control reviews [median (IQR), 6 (4, 7) compared to 3 (2, 5) respectively]. CAM reviews were less likely than control reviews to search either MEDLINE® or EMBASE® [68% (89/131) versus 98% (172/175), respectively], or to report hand-searching for literature (66% of CAM reviews versus 88%, respectively). However, CAM reviews were more likely to completely report dates of searching [86% (113/131) versus 35% (61/175) of control reviews].

#### Screening and data extraction

CAM reviews were more likely to have specified a primary outcome [57% (75/131) compared to 21% (37/175) control], and slightly more likely to have described the methods used in screening studies for inclusion [57% (75/131) of CAM SRs compared to 21% (37/175) of control sample SRs]. Almost one-third of both CAM and control SRs [31% (41/131) versus 29% (50/175), respectively] did not report how data extraction was carried out.

#### Review methods: assessing risk of bias

The risk of bias assessment within included studies varied considerably across the samples. For example, although 28% (37/131) of CAM SRs and 17% (30/175) of control SRs used the Cochrane Risk of Bias Tool [19], 83% of CAM reviews used a tool identified as relatively less frequently used [20] (e.g., MINORS [21], Downs and Black [22], Zaza [23]). Self-developed tools were used in 4% (5/131) of CAM reviews and 11% (19/175) of control SRs. Of the CAM reviews, 19/131 reviews used more than one tool (Table 3).

#### Results and discussion sections

More than half of all reviews included a PRISMA-like flow diagram [50% (66/131) of CAM SRs and 55% (96/175) of control SRs]. Heterogeneity or ‘consistency’ amongst included studies was formally assessed frequently across both groups. The CAM sample contained less than 5% (6/131) of SRs reporting an assessment for publication bias in comparison to a 46% (80/175) reporting rate in the control sample. The most common means of assessing publication bias was by funnel plot. Over 80% of reviews in both samples discussed the limitations of their review (82% CAM SRs and 90% control SRs). Source of funding was frequently and consistently underreported, and less than 5% of reviews across samples were reported as being funded by for-profit organisations (0% CAM SRs versus 3% control SRs) (Table 3).

## Discussion

Systematic reviews (SRs) are being published in abundance and, as such, their reporting characteristics and methodological rigor must be assessed to ensure that research produced is of the highest standard. Research in the field of CAM is considerable with 43,312 trials listed in the Cochrane CAM field trials database, and approximately 10% of Cochrane reviews are CAM-related, as of October 2012 [24]. Thus, it is important to independently assess the quality of reporting of CAM reviews and useful to draw comparisons to a more general sample of SRs to assess the strengths and weaknesses of both groups. Many findings of this evaluation are notable and suggest that there are some considerable differences between how CAM and control SRs are conducted and subsequently reported. There is no evident consistency in the completeness of reporting or quality of conduct between samples. As a result, findings should be considered on an item-by-item basis.

### Similarities between CAM and Control SRs

Many similarities in the frequency of adequate reporting between CAM and control SRs were observed. The number of reported updates was low across both samples, perhaps due to limited funding availability or other barriers [25]. Many reviews from both samples did not report the use of reporting guidelines to assist in report writing. This may imply that reporting guidelines were not followed or that guideline use was simply not reported. Selective reporting was not assessed sufficiently across both samples, perhaps due to a lack of available guidance for dealing with this potential bias. In analyses, a number of SRs across groups reported running both fixed and random effects models; again the guidance in this regard is not explicit about the appropriateness of such a measure. However, it is our recommendation that the model used should always be pre-specified and reported in a publicly available review protocol. Finally, source of funding was frequently and consistently underreported in both samples, possibly indicating an area of reporting that is in need of improvement across all SR research.

### Discrepancies between CAM and Control SRs

There were a number of discrepancies between both groups. CAM SRs were found to be published in journals with a lower median impact factor compared to the control sample. Also, the focus of CAM reviews was almost exclusively for evaluating treatments, whereas 15% of control reviews evaluated preventive therapies. This is not unexpected because preventative therapies typically require longer term and more expensive trials, there are limited resources to conduct such trials of CAM interventions which are typically not industry funded. In 68% of CAM reviews and 98% of control reviews, either MEDLINE or EMBASE, or both were searched. This is an interesting result, in that many reviewers consider it standard practice to search both MEDLINE and EMBASE. Therefore, it is surprising that 32% of CAM SRs did not search either database, regardless of the diverse nature of review topics that often require searching of less well known databases as well. Despite MEDLINE® and EMBASE® being searched less frequently in CAM reviews, on average, CAM reviews did search more databases; this is consistent with previous findings [26] and with the language-based hypothesis above. Risk of bias assessment within included studies varied considerably across the samples; 28% of CAM SRs and 17% of control SRs used the Cochrane Risk of Bias Tool [19]. These findings are consistent with other research [27]. Moreover, 83% of CAM reviews used less prominent tools and self-developed tools were used in 4% of CAM reviews and 11% of control SRs. There are a substantial number of methods used to assess the quality of primary studies in both samples of SRs. This is consistent with previous research which reported of 177 reviews, 38% defined a method of quality assessment, within which 74 different methodological items and 26 different scales were identified [21].

Assessment of publication bias was reported in 46% of the control sample reviews, compared to less than 5% of SRs in the CAM sample. Accepted methods for assessing publication bias recommend the inclusion of ten or more studies [28].Therefore, the less frequent assessment in CAM reviews could be explained by the 25% lower rate of less formal meta-analyses compared to control SRs, or potentially due to the inclusion of fewer primary studies in CAM reviews.

### Similarities and Differences between these and Previous Findings

In considering our findings in comparison to those of the Moher 2007 paper assessing 300 SRs (we refer to this as the ‘2004 sample’), we interpret these comparisons cautiously as there are some differences in sampling methods, most notably in the inclusion of Cochrane reviews in the 2004 sample. Similar to the comparison CAM and control samples in this evaluation, there are similarities and differences are between the 2004 sample and the current samples. Similarities include both the control sample and the 2004 sample having comparable frequencies with databases searched per review [median (IQR), 3 (2, 5)]. While over 65% of both CAM and control SRs used the term “systematic review” or “meta-analysis” in the title, this was the case for only 50% of the 2004 sample. The percentage of CAM reviews with reported primary outcomes was similar to that of the 2004 sample.

Considerable differences were noted in the frequency of reviews conducting cost-effectiveness analyses, with both the CAM and control samples having relatively low numbers compared to the 2004 sample. This is potentially due to the 2004 sample including more health technology assessments in which more cost-effectiveness analyses are generally conducted. The 2004 sample of reviews saw assessment of publication bias reported in 31% of review, while this item was reported more frequently in the control sample and less frequently in the CAM sample. The finding that less than 5% of all SRs reported public availability of a review protocol differs substantially from the 46% seen in the 2004 sample. This most likely reflects the impact of the large number of Cochrane reviews in the 2004 sample, which all require a published protocol. In the 2004 sample, 53.7% of reviews conducted meta-analysis; this number has increased to 75% in the control sample, whereas the findings for CAM remained consistent with the 2004 sample. Moreover, both current samples saw a smaller percentage of updated reviews compared to the 2004 sample.

Both the CAM and control samples had a higher number of multi-authored reports compared to the 2004 sample of reviews. We consider this to be positive, as participation of more authors may contribute more well-rounded insight into the conduct and reporting of research. The increase in use of flow diagrams in reports, the extent of consistency amongst included studies, and the completeness of reporting of review limitations have also increased in the collective 2011 sample, compared to the 2004 sample. The considerably higher frequency of reporting of a flow diagram in 2011 may suggest that the QUality Of Reports Of Meta-analyses of randomised controlled trials (QUOROM [29] and subsequent PRISMA [3] reporting guidelines are having an impact on the reporting of SRs.

### Limitations

There are some limitations to this evaluation. In particular, the magnitude of differences between the CAM and control SRs may be due to discrepancies in how the groups were sampled. Both 2011 strategies were modelled from the 2004 search strategy [4] however, some temporal variation could be present due to the time periods in which the samples were taken (2011 versus 2004). Further, the 2004 sample was restricted to English-language publications only, while we did not restrict the CAM sample to English-language reviews. Due to the size of the 2011 control SR sample yield, we restricted the search to core clinical journals. Applying this filter reduced the screening burden considerably (∼20,000 records) by focussing on journals which are deemed by the National Library of Medicine to be of immediate interest to practicing clinicians. There is no evidence to suggest that core clinical journals systematically differ from all other journals however, this may have had a minor influence on the results of this study. The evolution of the PRISMA Statement in 2009 [3], used to define a SR in this research, may have potentially resulted in a different population of eligible SRs in comparison to the 2004 sample, possibly affecting the comparison of frequencies between groups. The extent to which these selection criteria affect the results is unknown.

### Conclusion

In conclusion, the quality of reporting is variable between CAM and control SRs, and in comparison to the 2004 sample. The two 2011 samples exhibited different strengths and weaknesses, but no discernible patterns emerged. This could be explained by the possibility that, as a whole, CAM researchers may operate somewhat differently than the general research community, with different priorities and ways of conducting and reporting research, while still adhering to some of the basic principles of good reporting. The inconsistencies raise questions regarding the appropriateness and extent to which all reviews should aspire to report SR findings using the same systematic approach, or whether more specific reporting guidelines may be needed for specific research areas for SRs, such as CAM. Examples from other reporting guidelines, such as the CONSORT Statement [30], [31], suggest that extensions to particular subgroups are both feasible and warranted.

In some instances, there were similarities across one or more items between the two groups and/or between the 2011 and 2004 samples. This may indicate circumstances in which there is a need for continued improvements regarding particular aspects of reporting across all SR research. Educators and researchers focused on improving the quality of reporting of SRs may be able to use our finding to improve teaching, future research and the development and improvement of tools in this area. These findings may point to a need for more awareness and training on particular aspects of reporting quality that may be less of a priority among researchers in particular areas of research, or across all SRs. Future SRs would benefit from utilizing the PRISMA Statement [3], as it provides a useful and comprehensive tool for ensuring the quality of reporting when drafting SR reports.

## Supporting Information

Appendix S1 **Search Strategy.**
(RTF)
