# Supplementary material for: An Evaluation of Epidemiological and Reporting Characteristics of Complementary and Alternative Medicine (CAM) Systematic Reviews (SRs)
Source: PLoS One. 2013 Jan 14;8(1):e53536. doi: 10.1371/journal.pone.0053536 (PMC3544927; doi:10.1371/journal.pone.0053536)
Supplement: Appendix S1 — Search Strategy. (RTF) [file pone.0053536.s001.rtf]

Appendix S1:
Database: Ovid MEDLINE(R) without Revisions <1996 to May Week 3 2011>	
Search Strategy:			
--------------------------------------------------------------------------------		
1     exp alternative medicine/ (87641)			
2     osteopathic medicine/ (975)			
3     ((complement* or alternat* or osteopathic*) adj (therap* or medicine)).tw. (10970)	
4     exp Drugs, Chinese Herbal/ (16819)			
5     (Tai chi or qigong or qi gong or chi kung or chikung or yoga).tw. (1376)	
6     yoga/ (675)			
7     (reflexology or therapeutic touch or spiritual healing or faith healing or proprioceptive neuromuscular facilitation 
or effleurage or soft tissue manipulation or myofascial release or aston patterning or connective tissue massage or 
bidegewebsmassage or caring touch or hellerwork).tw. (791)	
8     (trager or lomilomi or lomi-lomi or tragerbowen therapy or bowen technique or neuromuscular therapy or 
bowen work or muscle energy technique$ or neuromuscular facilitation or (manual adj1 therap*)).tw. (749)	
9     (shiatsu or shiatzu or Chih Ya or Zhi Ya or massage or zero balancing or rolfing or zone therapy or structural integration 
or functional integration or alexander technique or fedendrais or acupressure or acupuncture or electroacupuncture or 
electro-acupuncture or trigger point$ or post isometric contract$ or contract relax or hold relax).tw. (11130)	
10     *relaxation/ (215)			
11     *sensory deprivation/ (754)			
12     restricted environmental stimulation therapy.tw. (0)		
13     *autogenic training/ (83)			
14     exp *hypnosis/ (1771)			
15     (iris diagnosis or iris diagnoses or vega test$3 or iridolog$ or shark cartilage).tw. (153)	
16     exp *tissue extracts/tu (344)			
17     *bees/ or *honey/ (3886)			
18     (hoxsey or gerson or doman delcato or unani or flower remed$3).tw. (119)		
19     vitalism/ (69)			
20     natural childbirth/ (895)			
21     balneotherapy/ or ammotherapy/ or ammotherapy/ or Steam Bath/ or mud therapy/ or hydrotherapy/ or Climatotherapy/ (1629)	
22     exp Transcutaneous Electric Nerve Stimulation/ (2973)		
23     diet, macrobiotic/ or vegetarianism/ or health food/ (2452)		
24     ion generators.tw. (5)			
25     *silymarin/tu (119)			
26     (agitated diluation$ or prayer$1 or applied kinesiology).tw. (982)		
27     or/1-26 (120555)			
28     meta analysis.pt. (25551)			
29     exp meta-analysis as topic/ (9125)			
30     (meta-analy* or metanaly* or metaanaly* or met analy* or integrative research or integrative review* or integrative overview* or 
research integration or research overview* or collaborative review*).ti,ab. (31128)	
31     (systematic review* or systematic overview* or technology assessment* or HTA or HTAs).ti,ab. (26622)	
32     exp Technology assessment, biomedical/ (5035)		
33     health technology assessment winchester england.jn. (632)		
34     (evidence report technology assessment or evidence report technology assessment summary).jn. (192)	
35     "cochrane database of systematic reviews".jn. (7068)		
36     or/28-35 (69319)			
37     limit 27 to "reviews (specificity)" (2170)			
38     27 and 36 (2405)			
39     37 or 38 (2803)			
40     39 (2803)			
41     limit 40 to yr="2010-2011" (391)			

			
1     meta analysis.pt. (29942)		
2     exp meta-analysis as topic/ (11627)		
3     (meta-analy* or metanaly* or metaanaly* or met analy* or integrative research or integrative 	
review* or integrative overview* or research integration or research overview* or collaborative 	
review*).ti,ab. (39396)		
4     (systematic review* or systematic overview* or technology assessment* or HTA or HTAs).ti,ab. 	
-33238			
5     exp Technology assessment, biomedical/ (8473)	
6     health technology assessment winchester england.jn. (662)	
7     (evidence report technology assessment or evidence report technology assessment summary).jn. 	
-194			
8     "cochrane database of systematic reviews".jn. (7752)	
9     or/1-8 (87773)		
10     limit 9 to yr="2010-2011" (18177)		
11     10 and ("201106$" or "201107$" or "201108$").ed. (2130)	
12     10 not 11 (16047)		
13     limit 12 to "core clinical journals (aim)" (1795)		
14     remove duplicates from 13 (1537)		
